# Entecavir for Patients with Hepatitis B Decompensated Cirrhosis in China: a meta-analysis

**DOI:** 10.1038/srep32722

**Published:** 2016-09-07

**Authors:** F.Y. Wang, B. Li, Y. Li, H. Liu, W.D. Qu, H.W. Xu, J.N. Qi, C.Y. Qin

**Affiliations:** 1 Shandong University, Shandong, 250100, China; 2 Department of Gastroenterology and Hepatology, Shandong Province Hospital Affiliated to Shandong University, Shandong, 250021, China; 3 Department of Intensive care unit, Qilu hospital affiliated to Shandong University, Shandong, 250012, China; 4 Department of Health, the Central Hospital of Tai’an, Shandong, 271000, China; 5 Central laboratory, Shandong Province Hospital Affiliated to Shandong University, Shandong, 250021, China

## Abstract

Evidence about the clinical effects of entecavir (ETV) for patients with hepatitis B decompensated cirrhosis remain controversial. Therefore, we perform this meta-analysis to assess the treatment outcomes of ETV in participants with hepatitis B decompensated cirrhosis. Relevant studies were identified by searching databases until the March 2016. A random-effects model was used to estimate summary relative risks (RRs) and 95% confidence intervals (CIs). GRADEprofiler3.6 was used to evaluate the quality of the evidence. A total of 26 studies (involving 2040 patients) were included. The quality of the evidence was classified from very low to high by the GRADED approach for all included RCTs. Meta-analysis showed that patients were more likely to experience HBV-DNA loss (RR:1.85, 95%CIs: 1.41 to 2.43, P < 0.0001 at 48 weeks), have normalized alanine aminotransferase levels (ALT) (P = 0.003 at 24 weeks, P = 0.02 at 48 weeks), and have a low mortality rate at 24 weeks (P = 0.003) when treated with ETV. There was no significant different between ETV and the control groups at the total mortality (P = 0.06) and HBeAg seroconversion (P = 0.14). In conclusion, ETV could be the first line therapy for patients with HBV related decompensated cirrhosis, because ETV could reduce the early mortality and move HBV DNA load down.

According to the latest clinical practice guidelines^1^ made by European association for the study of the liver(EASL), approximately one third of the world’s population has serological evidence of past or present infection with hepatitis B virus (HBV) and HBV-related end stage liver diseases or hepatocellular carcinoma(HCC) are responsible for over 0.5–1 million deaths per year. The guideline of prevention and treatment for chronic hepatitis (2015 version)^2^ of China points that about 7.18% of the population aged 1 to 59 years old in China are chronic HBV surface antigen (HBsAg) carriers, according to the epidemiological investigate nationwide. Now, there are about 0.093 billion people who are HBsAg carriers based on the epidemiological studies. Longitudinal studies of untreated patients with CHB indicate that, after diagnosis, the 5-year cumulative incidence of developing cirrhosis ranges from 8% to 20%. The 5-year cumulative incidence of hepatic decompensation is approximately 20% for untreated patients with compensated cirrhosis. Untreated patients with decompensated cirrhosis have a poor prognosis with a 14–35% probability of survival at 5 years^1^. Decompensated cirrhosis^3^ is characterized by significant abnormalities in liver functions, including raised serum bilirubin levels, a prolonged prothrombin time and/or the occurrence of complications such as ascites, hepatic encephalopathy and variceal bleeding. It is necessary for patients with hepatitis B decompensated liver diseases to be treated. Nucleos(t)ide analogue therapy is an important way to be used for decompensated liver according to the clinical treatment guideline. The patients treated with the deoxyguanosine nucleoside analogue ETV could achieve the HBV DNA suppression, the biochemical improvement and the histological improvement^4^. The efficacy of ETV for the treatment of patients with chronic hepatitis B was proved^5^, including patients with compensated liver disease^6^,^7^.

There were many studies on ETV used for patients with hepatitis B decompensated liver disease. Liaw *et al*. proved^8^ that patients generally had a good tolerance to ETV in the treatment of HBV related decompensated cirrhosis, ETV had a better virus response than adefovir dipivoxil (ADV), and mortality of patients with ETV was similar with lamivudine (LAM) by a randomized, open-label study. Yang J *et al*.^9^ thought that the HBV DNA level of ETV group reduced more than ADV group after a treatment of 48 weeks. Keating GM ^3^showed that patients had a significant liver function improvements from baseline after 12 months treatment of ETV in patients with decompensated cirrhosis.

There are several meta-analysis about ETV for patients with HBV related decompensated cirrhosis. For example, Peng H *et al*.^10^ only compared LAM combined with ADV with ETV. Ye X *et al*.^11^ just showed the effects of LAM and ETV for hepatitis B decompensated cirrhosis. Singal A. K. *et al*.^12^ did not study the difference of patients without anti-viral agents with patients who used ETV for HBV related decompensated liver cirrhosis. All records included in Singal A. K. *et al*.’s meta-analysis are published before 2010. In our review, 26 studies and 2040 patients are involved. We used the HBV DNA loss, ALT normalization, mortality and HBeAg seroconversion to evaluate the effect of ETV for patients with HBV related decompensated cirrhosis.

## Results

### Description of the included studies

**Figure 1:**
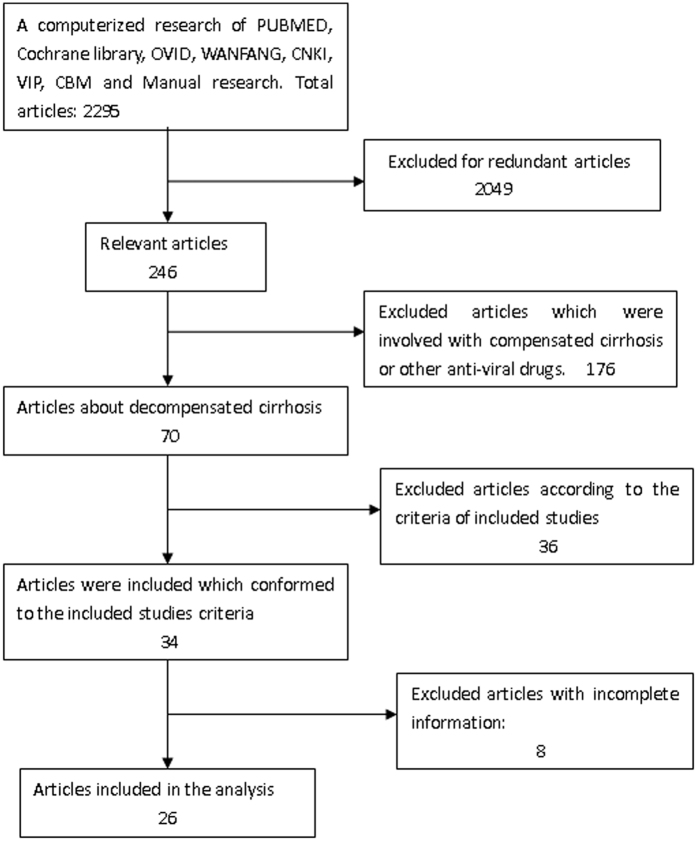
Flowchart of study inclusion protocol. Of the 2295 studies initially identified from our research, 26 met the inclusion criteria were included in this meta-analysis.

A total of 26 RCTs, with 2040 patients fulfilled the included criteria (Fig. 1 and Table 1). 930 patients were treated with ETV, 1110 patients were treated by other ways. The number of patients who were treated with other NAs drugs except ETV were 561, 549 patients were not treated by NAs drugs. The dominative outcomes are the HBV DNA loss, the recovery of ALT, the mortality and the HBeAg seroconversion.

### Risk of bias in included studies

**Figure 2:**
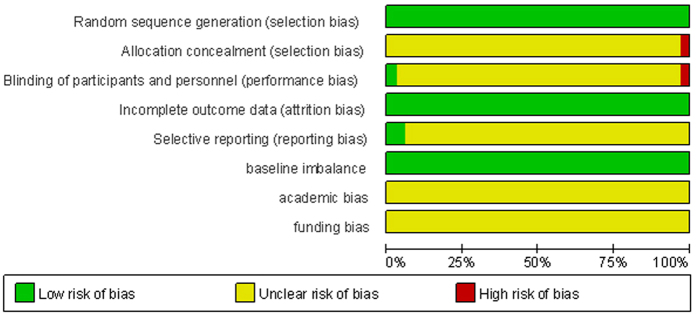
Risk of bias graph: review authors’ judgments about each risk of bias item presented as percentages across all included studies.

The summary results of the risk of bias were showed in Fig. 2. All trials were free from baseline imbalance bias and incomplete outcome bias. All trials were random control trials. None of the trials had adequate allocation concealment. One trial^13^ had adequate blinding. All trials might have academic bias and funding bias.

### Meta-analysis results

We used risk ratio (*RR*) as summary measures. We also stated the 95% confidence intervals.

### HBV DNA loss

#### HBV DNA loss at 12 weeks

**Figure 3:**
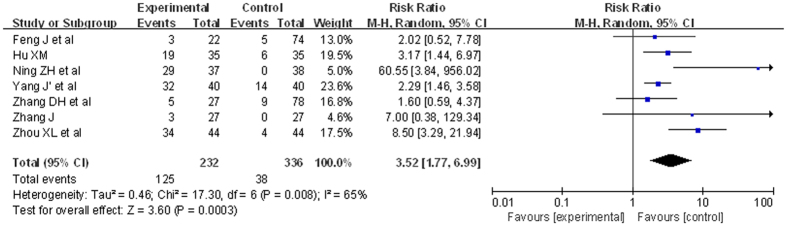
Comparison of ETV versus other treatments, outcome of HBV DNA loss at 12 weeks (forest plot). At 12 weeks, 232 patients treated with ETV moved more HBV DNA undetectable than 336 patients with other treatment. RR = 3.52, 95%CI [1.77, 6.99], P = 0.0003.

7 RCTs were included. There were 568 patients in total. The analysis of heterogeneity showed that *I*^*2*^ = 71%. These trials were considered statistically significant heterogeneity. We got *RR* = 3.52, 95%*CI* [1.77, 6.99], *P* = 0.0003. The experiment groups were higher than the control groups. This revealed a statistically significant. The result was showed in Fig. 3.

#### Subgroups of HBV DNA loss at 12w

There are four subgroups of HBV DNA loss in our research. Firstly, the control groups were patients with other NAs on the basis of CT.4 RCTs were included. There were 302 patients in total. The analysis of heterogeneity showed that *I*^*2*^ = 57%. These trials were considered statistically significant heterogeneity. We got *RR* = 4.37, 95%*CI* [1.58, 12.09], *P* = 0.004. The experiment groups were higher than the control groups. This revealed a statistically significant. Secondly, the control groups were patients with CT without any NAs. 3 RCTs were included. There were 217 patients in total. The analysis of heterogeneity showed that *I*^*2*^ = 31%. We got *RR* = 39.44, 95%*CI* [8.54, 182.03], *P* < 0.00001. The experiment groups were higher than the control groups. This revealed a statistically significant. Thirdly, the control groups were patients with LAM. 3 RCTs were included. There were 171 patients in total. The analysis of heterogeneity showed that *I*^*2*^ = 81%. These trials were considered statistically significant heterogeneity. We got *RR* = 1.23, 95%*CI* [0.23, 6.76], *P* = 0.81. There was no statistically significant between the experiment groups and the control groups. Fourthly, the control groups were patients with ADV only. 3 RCTs were included. There were 181 patients in total. The analysis of heterogeneity showed that *I*^*2*^ = 0%. These trials were no statistically significant heterogeneity. We got *RR* = 8.01, 95%*CI* [3.22, 19.94], *P* < 0.00001. The experiment groups were higher than the control groups. This revealed a statistically significant.

#### HBV DNA loss at 24 weeks

**Figure 4:**
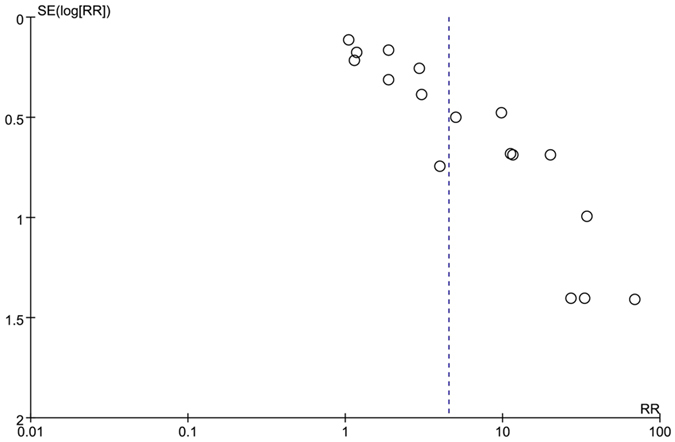
Funnel pool of comparison of ETV versus other treatments outcome of HBV DNA loss at 24 weeks.

17 RCTs were included. Total patients were 1324. The analysis of heterogeneity showed that *I*^*2*^ = 93%. These trials were considered statistically significant heterogeneity. We got *RR* = 4.51, 95%*CI* [2.51, 8.12], *P* < 0.00001. The experiment groups were higher than the control groups. This revealed a statistically significant. The result was showed in supplementary information. And the funnel pool was showed in Fig. 4.

#### Subgroups of HBV DNA loss at 24 weeks

There are four subgroups of HBV DNA loss at 24 weeks. Firstly, the control groups were patients with other NAs. 8 RCTs were included. Total patients were 689. The analysis of heterogeneity showed that *I*^*2*^ = 80%. These trials were considered statistically significant heterogeneity. We got *RR* = 1.64, 95%*CI* [1.16, 2.32], *P* = 0.005. There was statistically significant between the experiment groups and the control groups. Secondly, the control groups were patients without NAs drugs. 11 RCTs were included. Total patients were 684. The analysis of heterogeneity showed that *I*^*2*^ = 0%. We got *RR* = 13.04, 95%*CI* [7.99, 21.27], *P* < 0.00001. There was statistically significant between the experiment groups and the control groups. Thirdly, the control groups were patients with LAM. 4 RCTs were included. Total patients were 223. The analysis of heterogeneity showed that *I*^*2*^ = 57%. These trials were considered statistically significant heterogeneity. We got *RR* = 1.31, 95%*CI* [0.91, 1.86], *P* = 0.14. There was no statistically significant between the experiment groups and the control groups. Fourthly, the control groups were patients with ADV. 4 RCTs were included. Total patients were 372. The analysis of heterogeneity showed that *I*^*2*^ = 0%. We got *RR* = 3.13, 95%*CI* [2.1, 4.66], *P* < 0.00001. There was statistically significant between the experiment groups and the control groups.

#### HBV DNA loss at 48 weeks

14 RCTs were included. Total patients were 1195. The analysis of heterogeneity showed that *I*^*2*^ = 89%. These trials were considered statistically significant heterogeneity. We got *RR* = 1.85, 95%*CI* [1.41, 2.43], *P* < 0.0001. The experiment groups were higher than the control groups. This revealed a statistically significant. The result was showed in supplementary information.

#### Subgroups of HBV DNA loss at 48 weeks

There are four subgroups. Firstly, 10 RCTs were included. There were 845 patients. The analysis of heterogeneity showed that *I*^*2*^ = 80%. These trials were considered statistically significant heterogeneity. We got *RR* = 1.42, 95%*CI* [1.15, 1.74], *P* = 0.0009. The experiment groups were higher than the control groups. This revealed a statistically significant. Secondly, 7 RCTs were included. There were 449 patients. The analysis of heterogeneity showed that *I*^*2*^ = 96%. These trials were considered statistically significant heterogeneity. We got *RR* = 7.58, 95%*CI* [1.35, 42.59], *P* = 0.02. The experiment groups were higher than the control groups. This revealed a statistically significant. Thirdly, 5 RCTs were included. There were 280 patients. The analysis of heterogeneity showed that *I*^*2*^ = 68%. These trials were considered statistically significant heterogeneity. We got *RR* = 1.38, 95%*CI* [1.03, 1.86], *P* = 0.03. The experiment groups were higher than the control groups. This revealed a statistically significant. Fourthly, 5 RCTs were included. There were 307 patients. The analysis of heterogeneity showed that *I*^*2*^ = 49%. We got *RR* = 1.50, 95%*CI* [1.18, 1.89], *P* = 0.0007. The experiment groups were higher than the control groups. This revealed a statistically significant.

### ALT normalization

#### ALT normalization at 24 weeks

**Figure 5:**
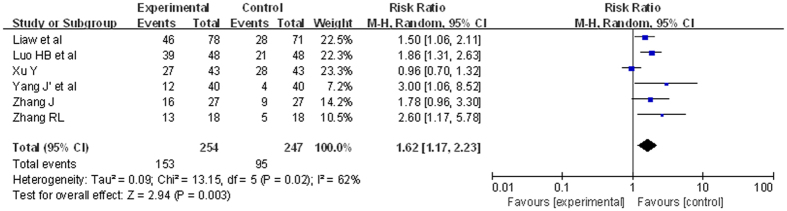
Comparison of ETV versus other treatments outcome of ALT normalization at 24 weeks (forest plot). At 24 weeks, 254 patients with ETV experienced more ALT normalization than 247 patients with other treatment. *RR* = 1.62, 95%*CI* [1.17, 2.23], *P* = 0.003.

6 RCTs were included. There were 501 patients. The analysis of heterogeneity showed that *I*^*2*^ = 62%. These trials were considered statistically significant heterogeneity. We got *RR* = 1.62, 95%*CI* [1.17, 2.23], *P* = 0.003. The experiment groups were higher than the control groups. This revealed a statistically significant. The result was showed in Fig. 5.

#### ALT normalization at 48 weeks

7 RCTs were included. Total patients were 622. The analysis of heterogeneity showed that *I*^*2*^ = 77%. These trials were considered statistically significant heterogeneity. We got *RR* = 1.38, 95%*CI* [1.06, 1.80], *P* = 0.02. There was statistically significant between the experiment groups and the control groups.

#### Mortality

**Figure 6:**
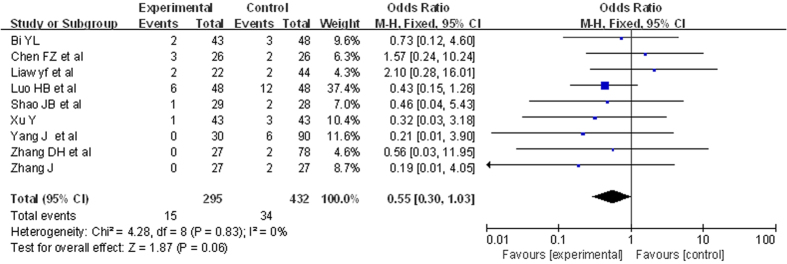
Comparison of ETV versus other treatments’ outcome of mortality. Subgroups of mortality (forest plot). The mortality of 295 patients treated with ETV did not show a statistically significant to 432 patients with other treatment. *RR* = 0.55, 95%*CI* [0.30, 1.03], *P* = 0.06.

9 RCTs were included. There were 727 patients. The analysis of heterogeneity showed that *I*^*2*^ = 0%. These trials were not considered statistically significant heterogeneity. We got *RR* = 0.55, 95%*CI* [0.30, 1.03], *P* = 0.06. This did not reveal a statistically significant. The result was showed in Fig. 6.

There are two subgroups of mortality. One subgroup was the mortality till 24 weeks.12 RCTs were included. There were 765 patients. The analysis of heterogeneity showed that *I*^*2*^ = 0%. These trials were not considered statistically significant heterogeneity. We got *RR* = 0.38, 95%*CI* [0.20, 0.71], *P* = 0.003. This revealed a statistically significant. The other was mortality till 48 weeks. 9 RCTs were included. There were 627 patients. The analysis of heterogeneity showed that *I*^*2*^ = 0%. These trials were not considered statistically significant heterogeneity. We got *RR* = 0.58, 95%*CI* [0.33, 1.03], *P* = 0.06. This did not reveal a statistically significant.

#### HBeAg seroconversion

**Figure 7:**
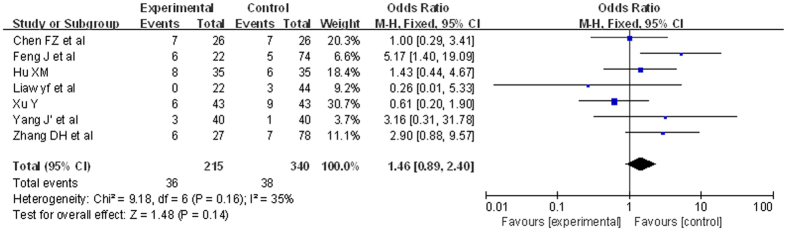
Comparison of ETV versus other treatments’ outcome of HBeAg seroconversion (forest plot). There were no significant difference about the rate of HBeAg seroconversion between ETV group and control group. *RR* = 1.46, 95%*CI* [0.89, 2.40], *P* = 0.14.

7 RCTs were included. There were 555 patients. The analysis of heterogeneity showed that *I*^*2*^ = 35%. These trials were not considered statistically significant heterogeneity. We got *RR* = 1.46, 95%*CI* [0.89, 2.40], *P* = 0.14. This did not reveal a statistically significant. The result was showed in Fig. 7.

#### Evidence quality

The results of the evidence quality were showed in supporting information.

## Discussion

ETV an oral deoxyguanosine nucleoside analogue, inhibits serum HBV DNA efficiently, improves the biochemical and histological characters of HBV related diseases^4^,^14^. S. Amini-Bavil-Olyaee *et al*.^15^ proved ETV was in short term a safe option for HBeAg negative patients. There were several meta-analysis^10^,^11^,^12^ about the oral anti-viral agents for patients with decompensated HBV related liver cirrhosis. However, there were not enough evidence to prove that ETV could be the first line drug for HBV related decompensated liver cirrhosis.

Chen FZ *et al*.^16^, Feng J *et al*.^17^, Zhang DH *et al*.^18^, Hu XM^19^ and Bi YL^20^ proved that the patients with ETV could undergo more HBV DNA loss than the patients with LAM at 24 weeks. In the studies of Shao JB *et al*.^21^, ETV made more HBV DNA loss than LAM at 12 weeks, but less at 24 weeks. Our data showed that ETV could significantly move viral load down to undetectable levels compared to patients without NAs treatment. The patients with ETV also experienced more HBV DNA loss than patients with ADV therapy. Although, at 12^th^ and 24^th^ weeks there were no significant differences in undetectable viral load between ETV and LAM in patients with Hepatitis B virus-related decompensated liver cirrhosis. ETV efficiently improve the outcome of HBV DNA loss than LAM at 48^th^ week. ETV’s long-term efficacy is superior to LAM at the part of HBV DNA loss.

ETV causes statistically significant sharp decline in ALT level at 24^th^ and 48^th^ week. Although, there were no significant differences between ETV and control groups at 48^th^ week.

Xu Y^22^ considered the mortality of ETV group was higher than the group with LDT and ADV. In our research, ETV reduces the mortality of patients at 24^th^ week. ETV could reduce early mortality.

Lin XS *et al*.^23^, Han ZQ *et al*.^24^, Yang J *et al*.^9^ and Feng J *et al*.^17^ thought ETV could improve the rate of HBeAg seroconversion. Liaw *et al*.^8^ thought HBeAg seroconversion was higher with ADV at 24 weeks. In our research, there were no significant different between the ETV group and the control group.

Liaw yf *et al*.^13^ thought there were no significant different between ETV and TDF among three treatment regimens(HBV DNA loss, ALT normalization and mortality), TDF was superior to ETV in terms of HBeAg seroconversion. Xu Y^22^ thought LDT combined with ADV early acting was better than ETV. However, there was only one paper to support their conclusion, more trials were needed.

We used Funnel pool to evaluate the publication bias and found that almost all the related meta-analysis had the publication bias. The results would be affected.

The degree of evidence quality about patients’ mortality (ETV versus other treatments) and HBV DNA loss (ETV versus patients only take CT) at 24 weeks was high. Night outcomes of the degree of evidence quality is moderate. Other results’ degrees were from very low to low. The low and very low quality of the evidence would affect the reliability of the results.

There are still some limits of our research. (i) We only evaluated four outcomes (the HBV DNA loss, the rates of ALT normalization, the mortality, the HBeAg seroconversion). Other results (such as pathological changes of liver tissue, cost-effectiveness issues are not mentioned in our study. (ii) There were no specific descriptions of the lower magnitude to the decline of detectable HBV DNA and the decrease of ALT. (iii) The number of RCTs included in this study is limited and the included samples’ number is insufficient. (iv) The quality of RCTs included in our study is not high. We still need RCTs of multi-center, high qualities and a large of samples to obtain a comprehensive Meta-analysis.

Despite the shortcomings of the studies included in our review, these studies constitute the best level of evidence that is currently available. Overall, the evidence from systematic review and meta-analysis is more trustworthy than observational studies and expert opinions. In our research, ETV could be the first line therapy for patients with HBV related decompensated cirrhosis, because ETV could reduce the early mortality and move HBV DNA load down.

## Methods

### Search method

A computerized search of The Cochrane Library (CENTRAL, 2016), PubMed (1966-March 2016), Embase (via OVID) (1974-March 2016), China National Knowledge Infrastructure (CNKI) (1978-March 2016), WANFANG (1998-March 2016), China Science and Technology Journal Database(VIP) (1989-March 2016), Chinese BioMedical Literature (CBM) (1978-March 2016) databases was conducted by two authors (WFY and LB) independently. We searched the terms of ETV, decompensated cirrhosis, hepatitis B, and randomized controlled trial. The results were limited by the MeSH terms of these words. Finally, we expanded the search results by the free word retrieval for the newest reports. All the citations of the identified trials were checked. We also checked the citations of published reviews meta-analysis or guidelines. Manual search was made to augment the search strategy.

### Criteria for considering studies for this review

All the included studies satisfied the following selection criteria: (i) types of studies-we included all randomized clinical trials, which compared the clinical effects of ETV with other nucleos(t)ide analogues (NAs) drugs or without other NAs drugs; (ii) types of participants-patients are older than 16 years who are diagnosed with HBV related decompensated liver cirrhosis according to the Management of chronic hepatitis B virus infection of China (2015); (iii) types of intervention – the experimental group: oral ETV; the control group: oral with/without other NAs drugs on the basis of comprehensive therapy (CT); (iv) types of outcome measures- HBV DNA loss, the level of serum alanine aminotransferase, the mortality, HBeAg seroconversion.

### Criteria of excluded studies

(i) repeat reports; (ii) design defect (eg. not a randomized controlled trial); (iii) incomplete data; (iv) co-infection with other viruses (eg. Hepatitis A virus); (v) other decompensated liver disease (eg. autoimmune liver disease).

### Assessment of risk of bias

We assessed the risk of bias in the trials by following the instructions given in Cochrane Handbook for Systematic Reviews of Interventions. We assessed the following procedures of each trials because the methodological quality of the trials could have an influence on intervention effects. We assessed the following parts: (i) random sequence generation (ii) allocation concealment (iii) blinding (iv) incomplete outcome data (v) selective outcome reporting (vi) baseline imbalance (vii) academic bias (viii) funding bias. Every domain was evaluated by three degrees which are low risk of bias, unclear risk of bias and high risk of bias.

### Subgroup analysis

We planned to perform the following subgroup analyses:

(i)  ETV versus other NAs.

(ii)  Trials with ETV versus trials only take CT without any antiviral drugs.

(iii) ETV versus LAM.

(iv)  ETV versus ADV.

### Statistical methods

We use the software package Review Manager 5.3.5 to perform the meta-analysis according to the recommendation of the Cochrane Collaboration^25^. We use risk ratio (RR) to calculate the 95% confidence interval for our research. In our research, all indices we included were dichotomous variables. We used a random-effects model for all studies. The random-effect model is DerSimonian-Laird. The heterogeneity was explored by chi-squared test with significance set at P value 0.10, and the quantity of heterogeneity^26^ was measured by *I*^*2*^. *I*^*2*^ < 50% was considered there was heterogeneity of the trials included. Generally, if *I*^*2*^ > 50% was considered statistically significant heterogeneity^27^.

We performed intention-to-treat analysis for the participants who could not finish the treatment. The patients who did not finish the treatment included patients who died, patients who gave up the treatment, and patients we could not connect with them. We considered these participants as negative results.

### Quality of the evidence

We used GRADEprofiler3.6 to evaluate the quality of the evidence according to the guideline of GRADES of Recommendations Assessment Development and Evaluation (GRADE). There are four degrees in GRADE: high, moderate, low and very low. The results of GRADE were showed by evidence profile (EP).

### Funnel plot

We intended to use funnel plot to measure the publication bias. Lau J *et al*.^28^ thought that at least 10 papers were needed for one funnel plot. In our research, we used funnel plot for studies which involved more than 10 essays.

## Additional Information

**How to cite this article**: Wang, F.Y. *et al*. Entecavir for Patients with Hepatitis B Decompensated Cirrhosis in China: a meta-analysis. *Sci. Rep.* **6**, 32722; doi: 10.1038/srep32722 (2016).

## Supplementary Material

Supplementary Information

## Figures and Tables

**Table 1:** Characteristics of included studies.

Study	Year	Number		Mean age	Intervention		Duration	Outcomes
		experiment	control	(years)	experiment	control	(weeks)	
					**(mg/day)**	**(mg/day)**		
Yang J *et al*.^29^	2012	30	30	50.7	0.5	ADV 10	48	A/B/C/D/E
			30	UC		LAM 100	48	A/B/C/D/E
			30	UC		CT	48	A/B/C/D/E
Lin XS *et al*.^23^	2011	32	32	49.7	0.5	CT	72	A/E/F
Ren WX *et al*.^30^	2014	27	27	48	0.5	CT	48	A/B/D/E
Luo HB *et al*.^31^	2009	48	48	54.9	0.5	CT	48	A/B
Ning ZH *et al*.^32^	2009	37	38	47.5	0.5	CT	24	A/B/F
Han ZQ *et al*.^24^	2009	30	30	56.2	0.5	CT	24	A/B/D/E/F
Chen FZ *et al*.^16^	2010	26	26	48.5	0.5	LAM 100	48	A/B/F
Zhang FL^33^	2011	28	26	45.6	0.5	CT	24	A/B/D
Zhang RL^34^	2010	18	18	UC	0.5	CT	24	A/B/E/F
Guo YM *et al*.^35^	2014	42	42	UC	0.5	CT	24	A/B/D/E
Yang J *et al*.^9^	2012	40	40	46.6	0.5	ADV 10	48	A/B/E/F
Li H^36^	2009	20	20	UC	0.5	CT	24	A/B/D/E
Xu Y^22^	2013	43	43	50.1	0.5	LDT 600 and ADV10	48	A/B/D
Feng J *et al*.^37^	2008	22	25	UC	0.5	LAM 100	48	A/B/D/F
			25	UC		ADV10	48	A/B/D/F
			24	UC		CT	48	A/B/D/F
Zhang DH *et al*.^18^	2011	27	27	UC	0.5	LAM 100	48	A/B/D/F
			27	UC		ADV 10	48	A/B/D/F
			24	UC		CT	48	A/B/D/F
Liaw *et al*.^8^	2011	100	91	51	1	ADV 10	96	A/B/F
Liaw yf *et al*.^13^	2011	22	45	54	0.5or1	TDF 300	48	A/B/F
Yang L^38^	2015	40	40	UC	0.5	LAM 100	48	C/E
Gulizire•Maola^39^	2015	35	35	UC	0.5	CT	48	A/B/C/D
Hu XM^19^	2014	36	36	46.5	0.5	LAM 100	1 year	A/B/C/F
Zhang J^40^	2014	27	27	UC	0.5	CT	48	A/B
Zhao ZY^41^	2014	36	36	UC	0.5	CT	48	A/B/E
Li MX^42^	2014	48	48	UC	0.5	CT	48	A/B/G
Zhou XL *et al*.^43^	2015	44	44	UC	0.5	CT	24	A/B/C/E
Bi YL^20^	2014	43	48	UC	0.5	LAM 100 and ADV 10	48	A/B/C/D
Shao JB *et al*.^21^	2010	29	28	43.6	0.5	LAM 100	96	A/E

UC: unclear; CT: comprehensive therapy (patients who did not use any NAs); A: HBV DNA; B: Hepatic function; C: Adverse Drug Reaction; D: Mortality; E: Child-pugh; F: HBeAg seroconversion; G: hepatitis B virus mutation rate.
