# Supplementary material for: Entecavir for Patients with Hepatitis B Decompensated Cirrhosis in China: a meta-analysis
Source: Sci Rep. 2016 Sep 7;6:32722. doi: 10.1038/srep32722 (PMC5013325; doi:10.1038/srep32722)
Supplement: Supplementary Information [file srep32722-s1.doc]

**Entecavir for Patients with Hepatitis B Decompensated Cirrhosis in China: a meta-analysis**

**Wang FY^1^, Li B^2^ ,Li Y^3^, Liu H^2^, Qu WD^4^, Xu HW^2^,Qi JN^5^, Qin CY^2＊^**

^1^ Shandong University, Shandong, 250100, China.

^2^ Department of Gastroenterology and Hepatology, Shandong Province Hospital Affiliated to Shandong University, Shandong, 250021, China.

^3^ Department of Intensive care unit, Qilu hospital affiliated to Shandong University, Shandong, 250012, China.

^4^ Department of Health, the Central Hospital of Tai’an, Shandong, 271000, China.

^5^ Central laboratory, Shandong Province Hospital Affiliated to Shandong University, Shandong, 250021, China.

^*^ Correspondence to : Prof. Qin CY, Department of Gastroenterology and Hepatology, Shandong Province Hospital, No.324, Jingwuweiqi Road,Shandong, TEL: 15165153211, China. E-mail: [qinchengyong@hotmail.com](mailto:qinchengyong@hotmail.com)

Supplementary information

Additional supporting information

Figure 1. Subgroups of HBV DNA loss at 12w

Figure 2. Funnel pool of subgroups of HBV DNA loss at 12w

Figure 3. Comparison of ETV versus other treatments outcome of HBV DNA loss at 24 weeks

Figure 4. Subgroups of HBV DNA loss at 24w

Figure 5. Funnel pool of subgroups of HBV DNA loss at 24w

Funnel pool of subgroups of HBV DNA loss at 48w

Figure 6. Comparison of ETV versus other treatments,outcome of HBV DNA loss at 48 weeks

Figure 7. Subgroups of HBV DNA loss at 48w Figure 8.

Figure 9. Subgroups of comparison of ETV versus other treatments' outcome of mortality at 48 weeks

Figure 10. Comparison of ETV versus other treatments,outcome of ALT normalization at 48w

Figure 11. Funnel pool of subgroups of mortality at 48w

Figure 12. Risk of bias summary: Review authors’ judgments about each risk of bias item for each included study

Table 1. Entecavir for hepatitis B decompensated cirrhosis in China


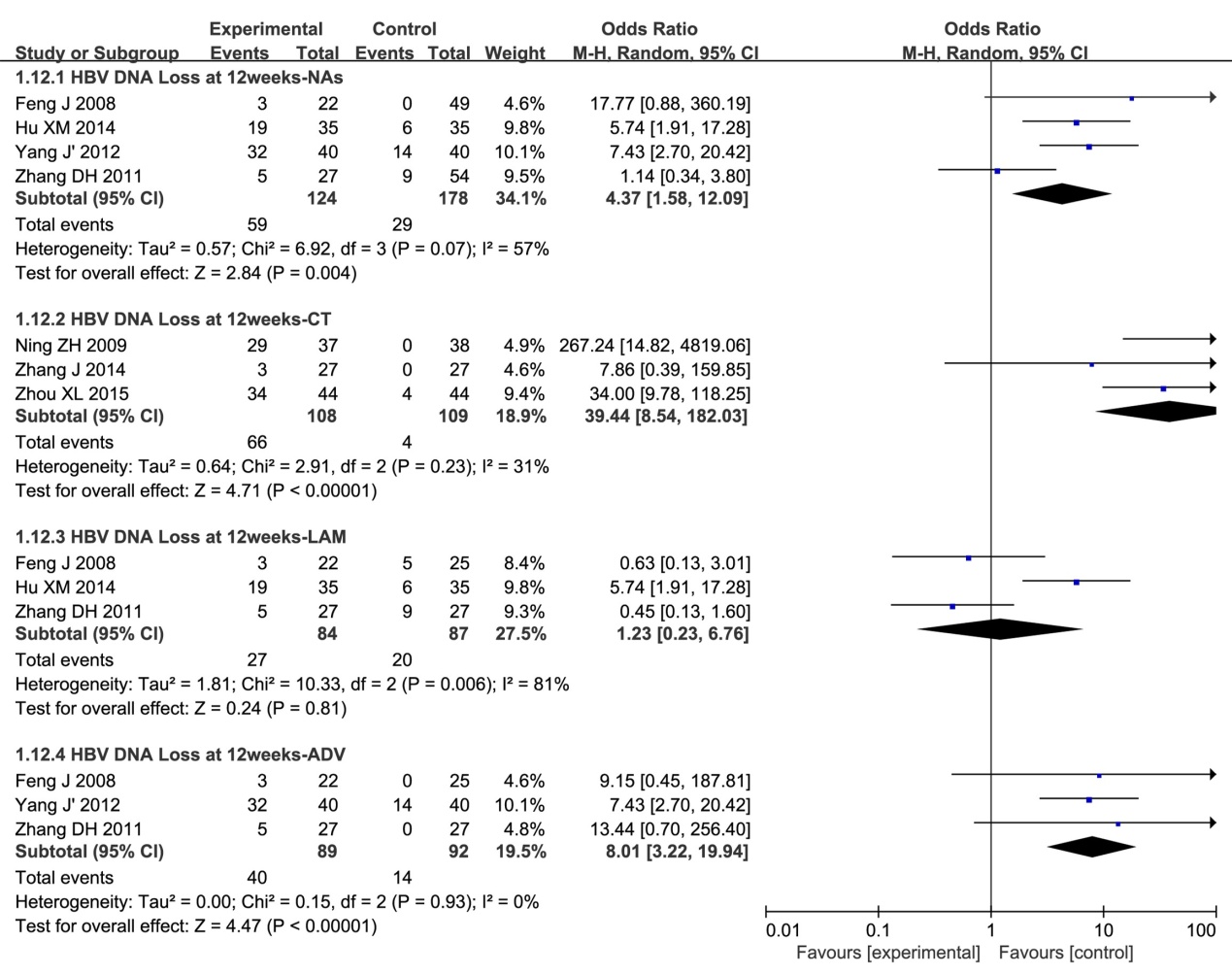


Figure 1. Subgroups of HBV DNA loss at 12w


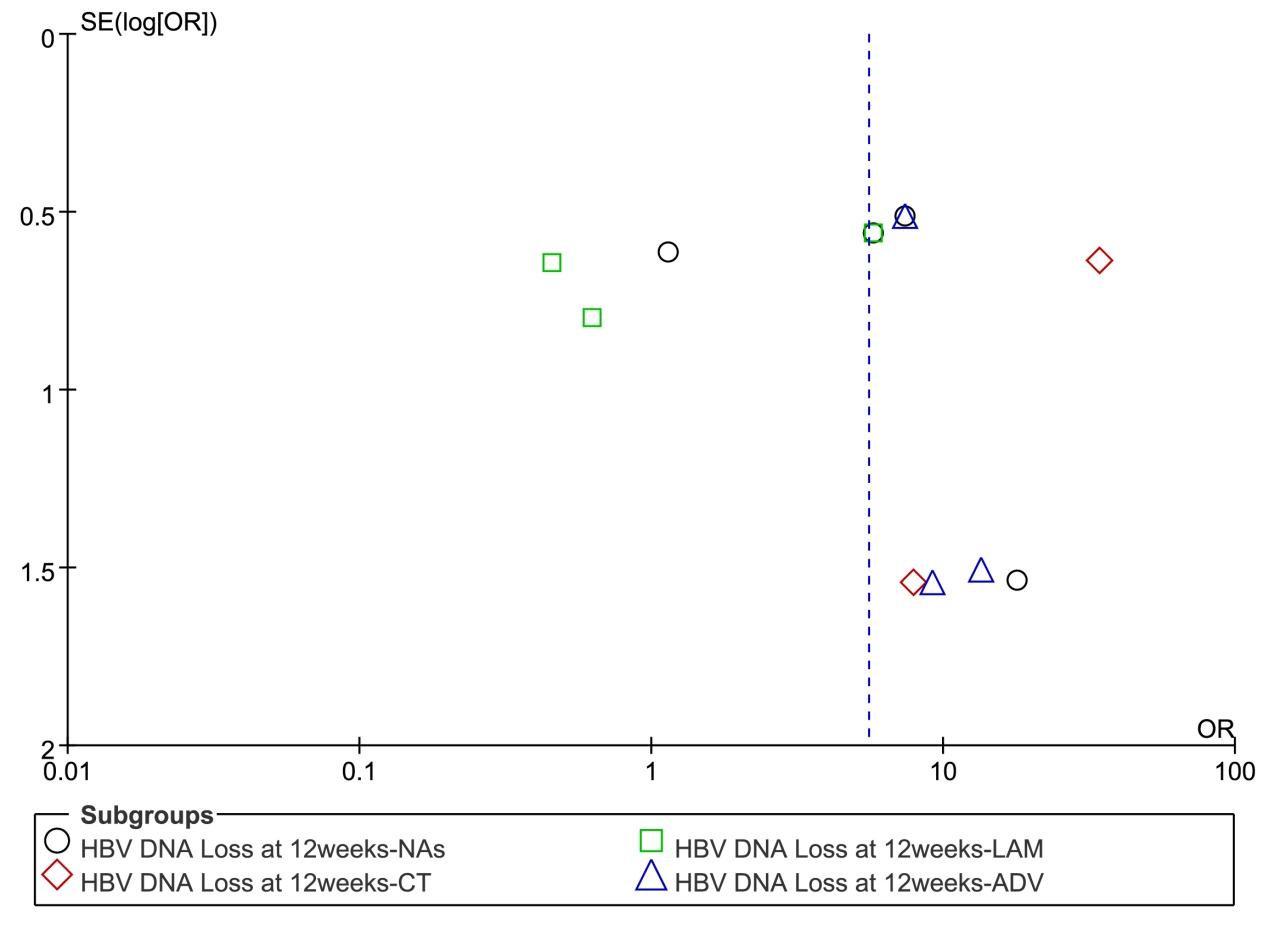


Figure 2. Funnel pool of subgroups of HBV DNA loss at 12w

Figure 3. Comparison of ETV versus other treatments outcome of HBV DNA loss at 24 weeks


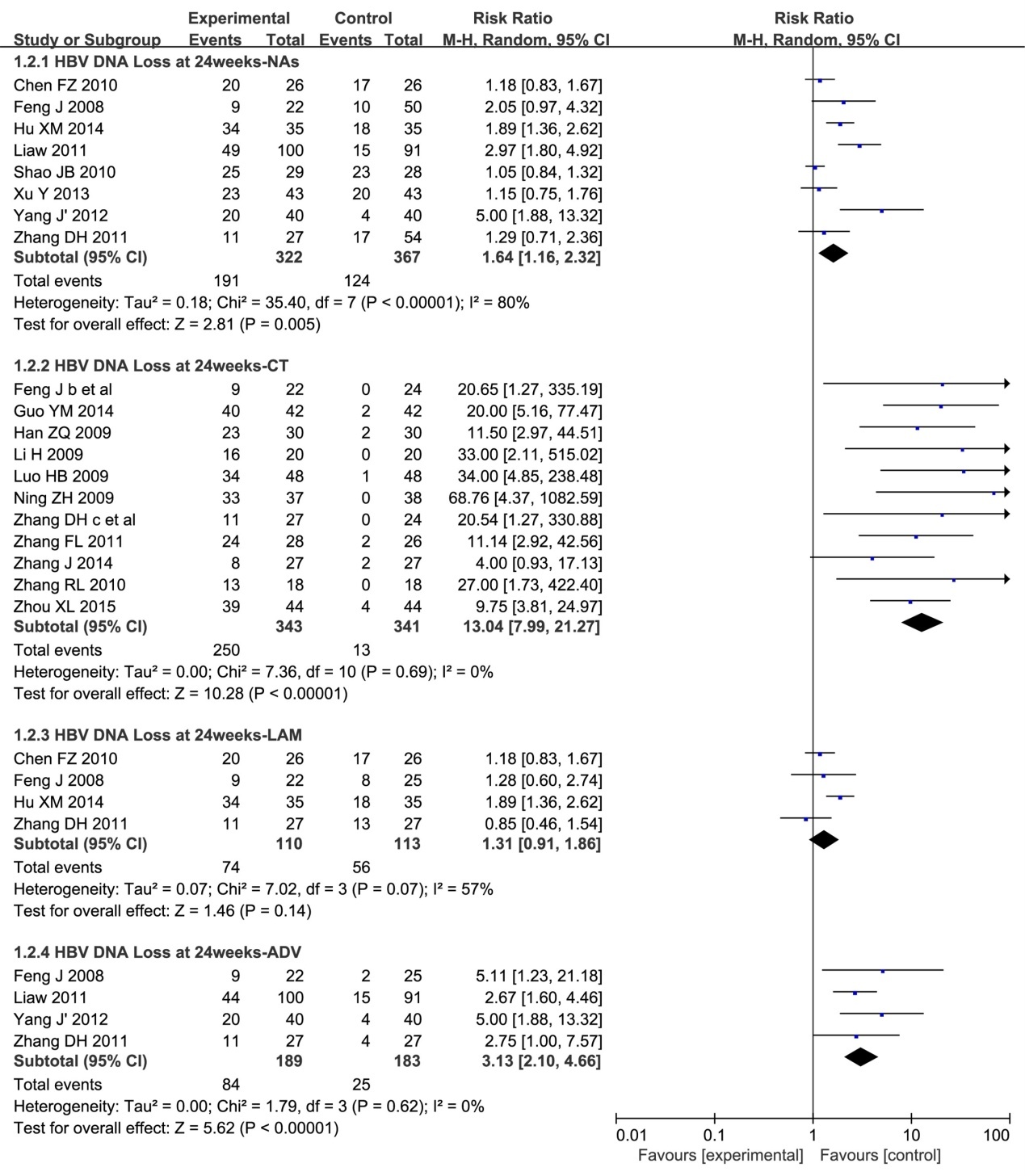


Figure 4. Subgroups of HBV DNA loss at 24w


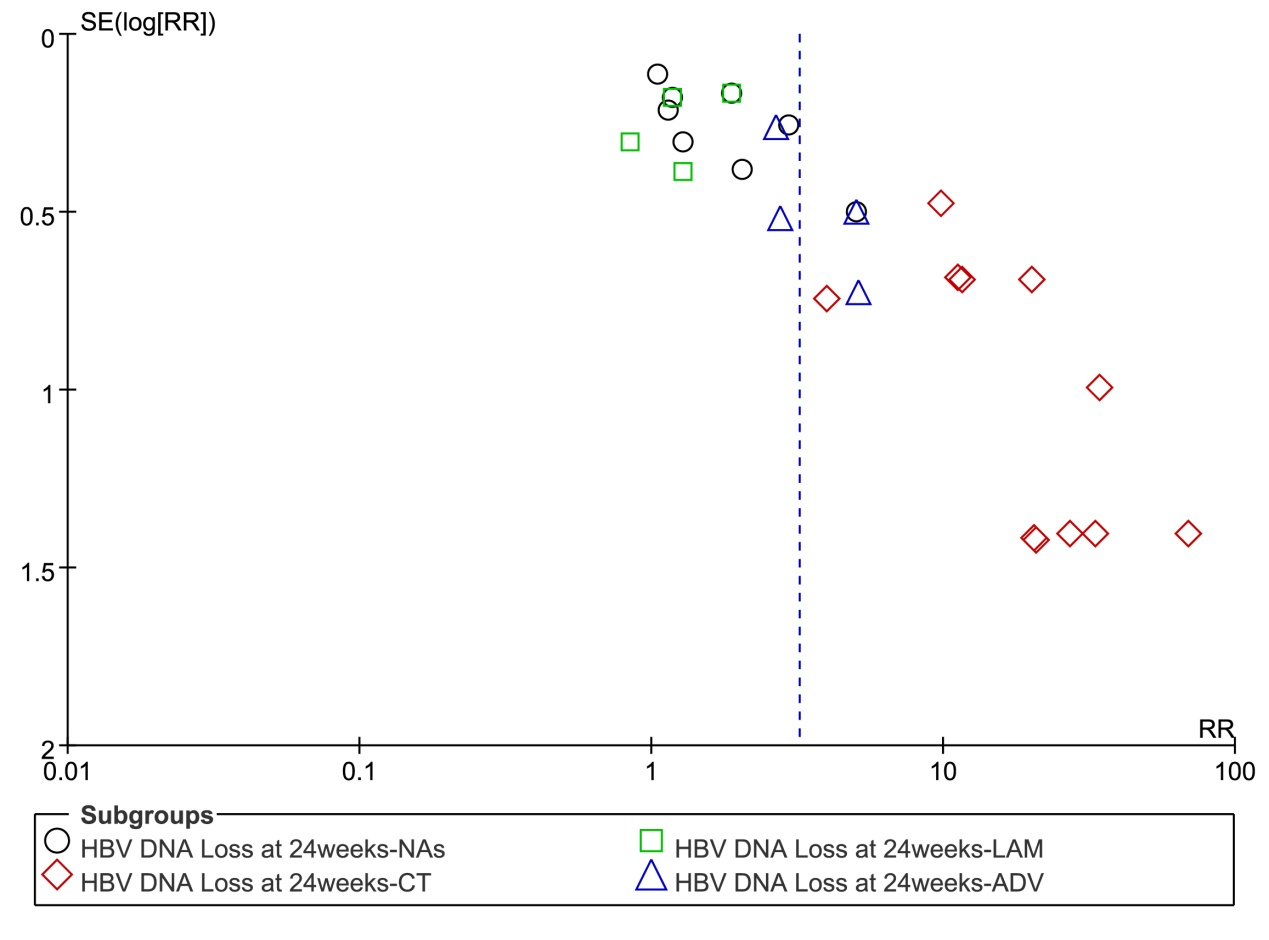


Figure 5. Funnel pool of subgroups of HBV DNA loss at 24w

Figure 6. Comparison of ETV versus other treatments,outcome of HBV DNA loss at 48 weeks.


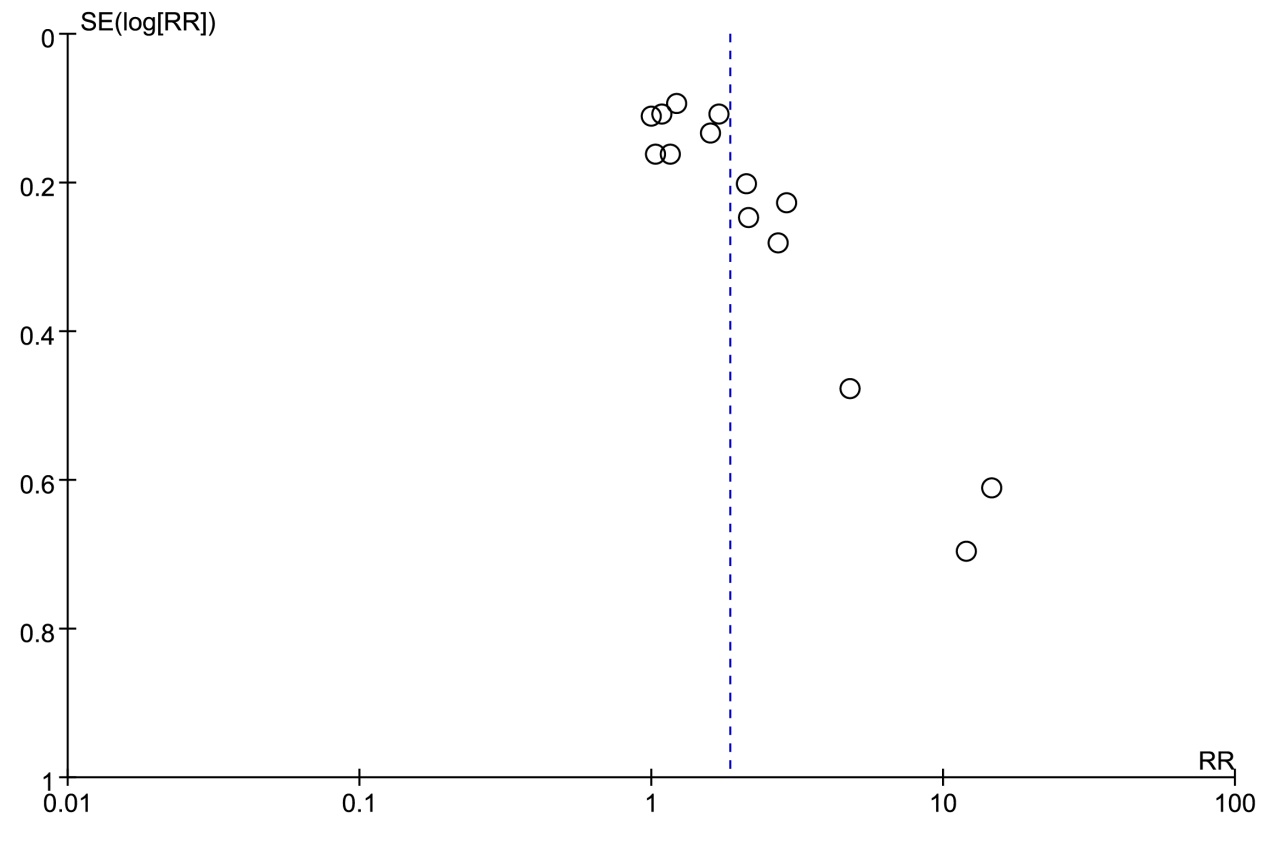


Figure 7. Funnel pool of subgroups of HBV DNA loss at 48w


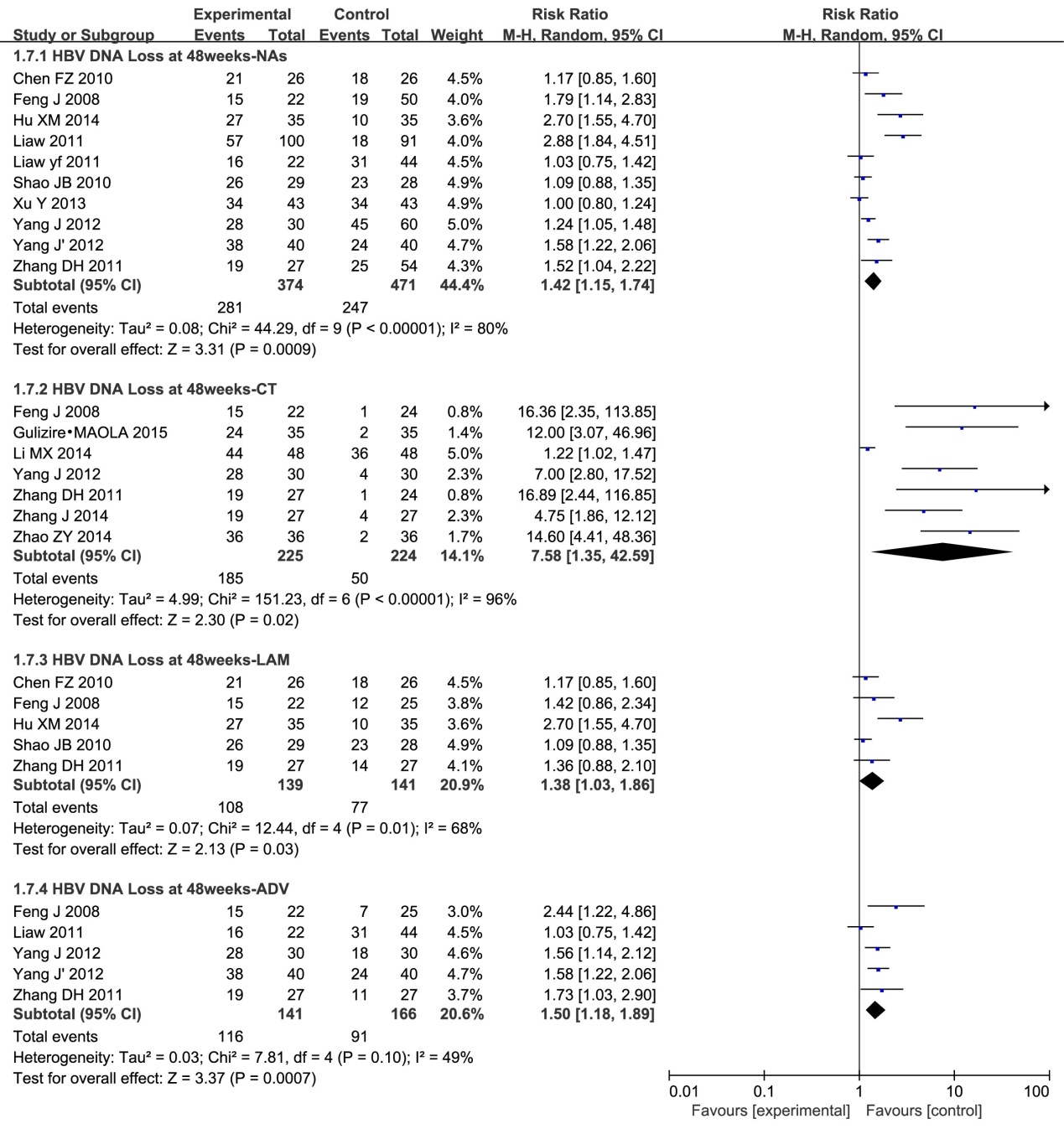


Figure 8. Subgroups of HBV DNA loss at 48w


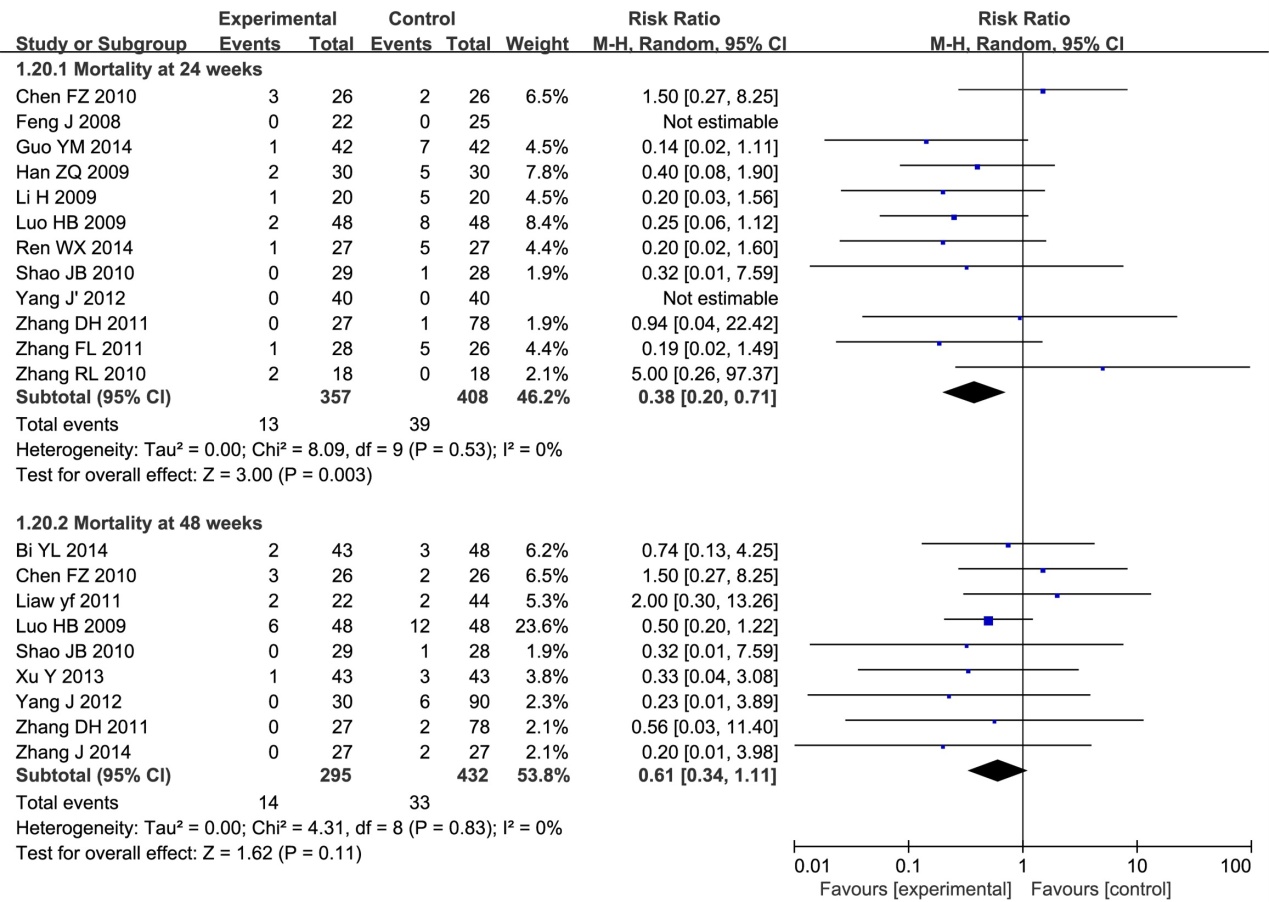


Figure 9. Subgroups of comparison of ETV versus other treatments' outcome of mortality at 48 weeks

Figure 10. Comparison of ETV versus other treatments,outcome of ALT normalization at 48w.


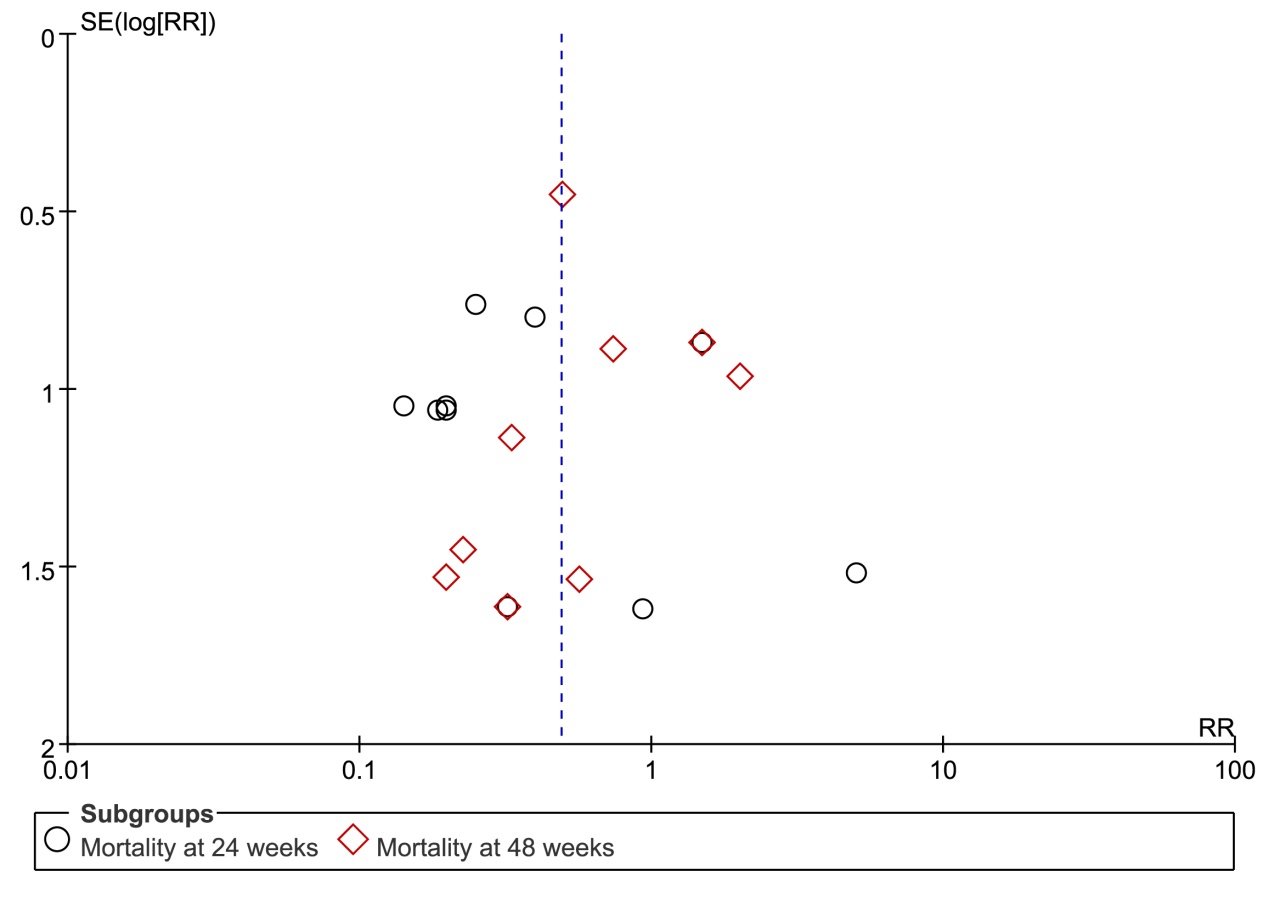


Figure 11. Funnel pool of subgroups of mortality at 48w

Figure 12. Risk of bias summary: Review authors’ judgments about each risk of bias item for each included study.

| **Table 1︱Entecavir for hepatitis B decompensated cirrhosis in China** | | | | | |
| --- | --- | --- | --- | --- | --- |
| **Bibliography:** . entecavir for hepatitis B decompensated cirrhosis 2016]. | | | | | |
| **Outcomes** | **No of Participants (studies)** Follow up | **Quality of the evidence** (GRADE) | **Relative effect (95% CI)** | **Anticipated absolute effects** | |
|  |  |  |  |  |  |
|  |  |  |  | **Risk with Control** | **Risk difference with Entecavir for hepatitis B decompensated cirrhosis** (95% CI) |
| **HBV DNA Loss at 12 weeks** | 568 (7 studies) 12 weeks | ⊕⊕⊕⊝ **MODERATE**^1,2,3^ due to risk of bias, inconsistency, large effect | **RR 3.52**  (1.77 to 6.99) | **Study population** | |
|  |  |  |  | **113 per 1000** | **285 more per 1000** (from 87 more to 677 more) |
|  |  |  |  | **Moderate** | |
|  |  |  |  | **91 per 1000** | **229 more per 1000** (from 70 more to 545 more) |
| **HBV DNA Loss at 12 weeks-NAs** | 871 (7 studies) 12 weeks | ⊕⊕⊕⊝ **MODERATE**^1,2,3^ due to risk of bias, inconsistency, large effect | **OR 5.52**  (2.4 to 12.67) | **Study population** | |
|  |  |  |  | **144 per 1000** | **337 more per 1000** (from 143 more to 536 more) |
|  |  |  |  | **Moderate** | |
|  |  |  |  | **167 per 1000** | **358 more per 1000** (from 158 more to 551 more) |
| **HBV DNA Loss at 12 weeks-CT** | 217 (3 studies) 12 weeks | ⊕⊕⊕⊝ **MODERATE**^1,2,4,5^ due to risk of bias, inconsistency, imprecision, large effect | **RR 13.49**  (5.7 to 31.92) | **Study population** | |
|  |  |  |  | **37 per 1000** | **458 more per 1000** (from 172 more to 1000 more) |
|  |  |  |  | **Moderate** | |
|  |  |  |  | **0 per 1000** | **-** |
| **HBV DNA loss at 12 weeks-LAM** | 171 (3 studies) 12 weeks | ⊕⊝⊝⊝ **VERY LOW**^1,2,4^ due to risk of bias, inconsistency, imprecision | **RR 1.38**  (0.83 to 2.29) | **Study population** | |
|  |  |  |  | **230 per 1000** | **87 more per 1000** (from 39 fewer to 297 more) |
|  |  |  |  | **Moderate** | |
|  |  |  |  | **200 per 1000** | **76 more per 1000** (from 34 fewer to 258 more) |
| **HBV DNA Loss at 12 weeks-ADV** | 181 (3 studies) 12 weeks | ⊕⊕⊝⊝ **LOW**^1,2,3,4^ due to risk of bias, inconsistency, imprecision, large effect | **RR 2.75**  (1.75 to 4.34) | **Study population** | |
|  |  |  |  | **152 per 1000** | **266 more per 1000** (from 114 more to 508 more) |
|  |  |  |  | **Moderate** | |
|  |  |  |  | **0 per 1000** | **-** |
| **HBV DNA Loss at 24 weeks** | 1324 (17 studies) 24 weeks | ⊕⊕⊕⊝ **MODERATE**^1,2,3^ due to risk of bias, inconsistency, large effect | **RR 4.51**  (2.51 to 8.12) | **Study population** | |
|  |  |  |  | **194 per 1000** | **679 more per 1000** (from 292 more to 1000 more) |
|  |  |  |  | **Moderate** | |
|  |  |  |  | **91 per 1000** | **319 more per 1000** (from 137 more to 648 more) |
| **HBV DNA Loss at 24 weeks-NAs** | 1968 (19 studies) 24 weeks | ⊕⊕⊝⊝ **LOW**^1,2^ due to risk of bias, inconsistency | **RR 3.19**  (2.18 to 4.67) | **Study population** | |
|  |  |  |  | **217 per 1000** | **476 more per 1000** (from 256 more to 797 more) |
|  |  |  |  | **Moderate** | |
|  |  |  |  | **100 per 1000** | **219 more per 1000** (from 118 more to 367 more) |
| **HBV DNA Loss at 24 weeks-CT** | 684 (11 studies) 24 weeks | ⊕⊕⊕⊕ **HIGH**^1,4,5^ due to risk of bias, imprecision, large effect | **RR 13.04**  (7.99 to 21.27) | **Study population** | |
|  |  |  |  | **38 per 1000** | **459 more per 1000** (from 266 more to 773 more) |
|  |  |  |  | **Moderate** | |
|  |  |  |  | **21 per 1000** | **253 more per 1000** (from 147 more to 426 more) |
| **HBV DNA loss at 24 weeks-LAM** | 223 (4 studies) 24 weeks | ⊕⊝⊝⊝ **VERY LOW**^1,2,4^ due to risk of bias, inconsistency, imprecision | **OR 2.15**  (1.22 to 3.8) | **Study population** | |
|  |  |  |  | **496 per 1000** | **183 more per 1000** (from 50 more to 293 more) |
|  |  |  |  | **Moderate** | |
|  |  |  |  | **498 per 1000** | **183 more per 1000** (from 50 more to 292 more) |
| **HBV DNA loss at 24 weeks-ADV** | 372 (4 studies) 24 weeks | ⊕⊕⊕⊝ **MODERATE**^1,3,4^ due to risk of bias, imprecision, large effect | **OR 4.99**  (3 to 8.3) | **Study population** | |
|  |  |  |  | **137 per 1000** | **305 more per 1000** (from 185 more to 431 more) |
|  |  |  |  | **Moderate** | |
|  |  |  |  | **124 per 1000** | **290 more per 1000** (from 174 more to 416 more) |
| **HBV DNA Loss at 48weeks** | 1195 (14 studies) 48 weeks | ⊕⊕⊝⊝ **LOW**^1,2^ due to risk of bias, inconsistency | **RR 1.85**  (1.41 to 2.43) | **Study population** | |
|  |  |  |  | **436 per 1000** | **370 more per 1000** (from 179 more to 623 more) |
|  |  |  |  | **Moderate** | |
|  |  |  |  | **439 per 1000** | **373 more per 1000** (from 180 more to 628 more) |
| **HBV DNA Loss at 48weeks-NAs** | 1881 (14 studies) 48 weeks | ⊕⊕⊝⊝ **LOW**^1,2^ due to risk of bias, inconsistency | **RR 1.72**  (1.43 to 2.07) | **Study population** | |
|  |  |  |  | **464 per 1000** | **334 more per 1000** (from 200 more to 497 more) |
|  |  |  |  | **Moderate** | |
|  |  |  |  | **480 per 1000** | **346 more per 1000** (from 206 more to 514 more) |
| **HBV DNA Loss at 48 weeks-CT** | 449 (7 studies) 48 weeks | ⊕⊕⊕⊝ **MODERATE**^1,2,4,5^ due to risk of bias, inconsistency, imprecision, large effect | **RR 3.66**  (2.87 to 4.68) | **Study population** | |
|  |  |  |  | **223 per 1000** | **594 more per 1000** (from 417 more to 821 more) |
|  |  |  |  | **Moderate** | |
|  |  |  |  | **57 per 1000** | **152 more per 1000** (from 107 more to 210 more) |
| **HBV DNA Loss at 48 weeks-LAM** | 280 (5 studies) 48 weeks | ⊕⊕⊝⊝ **LOW**^1,2^ due to risk of bias, inconsistency | **OR 3.01**  (1.77 to 5.11) | **Study population** | |
|  |  |  |  | **546 per 1000** | **238 more per 1000** (from 134 more to 314 more) |
|  |  |  |  | **Moderate** | |
|  |  |  |  | **519 per 1000** | **246 more per 1000** (from 137 more to 327 more) |
| **HBV DNA Loss at 48 weeks-ADV** | 307 (5 studies) 48 weeks | ⊕⊕⊕⊝ **MODERATE**^1^ due to risk of bias | **RR 1.52**  (1.29 to 1.79) | **Study population** | |
|  |  |  |  | **548 per 1000** | **285 more per 1000** (from 159 more to 433 more) |
|  |  |  |  | **Moderate** | |
|  |  |  |  | **600 per 1000** | **312 more per 1000** (from 174 more to 474 more) |
| **ALT Normalization at 24weeks** | 501 (6 studies) 24 weeks | ⊕⊕⊝⊝ **LOW**^1,2^ due to risk of bias, inconsistency | **RR 1.62**  (1.17 to 2.23) | **Study population** | |
|  |  |  |  | **385 per 1000** | **238 more per 1000** (from 65 more to 473 more) |
|  |  |  |  | **Moderate** | |
|  |  |  |  | **364 per 1000** | **226 more per 1000** (from 62 more to 448 more) |
| **ALT Normalization at 48weeks** | 622 (7 studies) 48 weeks | ⊕⊕⊝⊝ **LOW**^1,2^ due to risk of bias, inconsistency | **RR 1.38**  (1.06 to 1.8) | **Study population** | |
|  |  |  |  | **517 per 1000** | **197 more per 1000** (from 31 more to 414 more) |
|  |  |  |  | **Moderate** | |
|  |  |  |  | **465 per 1000** | **177 more per 1000** (from 28 more to 372 more) |
| **Mortality total** | 727 (9 studies) 96 weeks | ⊕⊕⊕⊝ **MODERATE**^1^ due to risk of bias | **OR 0.55**  (0.3 to 1.03) | **Study population** | |
|  |  |  |  | **79 per 1000** | **34 fewer per 1000** (from 54 fewer to 2 more) |
|  |  |  |  | **Moderate** | |
|  |  |  |  | **70 per 1000** | **30 fewer per 1000** (from 48 fewer to 2 more) |
| **Mortality at 24 weeks** | 1492 (17 studies) 24 weeks | ⊕⊕⊕⊕ **HIGH**^1,3^ due to risk of bias, large effect | **RR 0.49**  (0.32 to 0.76) | **Study population** | |
|  |  |  |  | **86 per 1000** | **44 fewer per 1000** (from 21 fewer to 58 fewer) |
|  |  |  |  | **Moderate** | |
|  |  |  |  | **70 per 1000** | **36 fewer per 1000** (from 17 fewer to 48 fewer) |
| **Mortality at 48 weeks** | 727 (9 studies) 48 weeks | ⊕⊕⊕⊝ **MODERATE**^1^ due to risk of bias | **RR 0.58**  (0.33 to 1.03) | **Study population** | |
|  |  |  |  | **76 per 1000** | **32 fewer per 1000** (from 51 fewer to 2 more) |
|  |  |  |  | **Moderate** | |
|  |  |  |  | **67 per 1000** | **28 fewer per 1000** (from 45 fewer to 2 more) |
| **HBeAg seroconversion** | 555 (7 studies) 48 weeks | ⊕⊕⊝⊝ **LOW**^1,4^ due to risk of bias, imprecision | **OR 1.46**  (0.89 to 2.4) | **Study population** | |
|  |  |  |  | **112 per 1000** | **43 more per 1000** (from 11 fewer to 120 more) |
|  |  |  |  | **Moderate** | |
|  |  |  |  | **90 per 1000** | **36 more per 1000** (from 9 fewer to 102 more) |
| *The basis for the **assumed risk** (e.g. the median control group risk across studies) is provided in footnotes. The **corresponding risk** (and its 95% confidence interval) is based on the assumed risk in the comparison group and the **relative effect** of the intervention (and its 95% CI). **CI:** Confidence interval; **RR:** Risk ratio; **OR:** Odds ratio; | | | | | |
| GRADE Working Group grades of evidence **High quality:** Further research is very unlikely to change our confidence in the estimate of effect.  **Moderate quality:** Further research is likely to have an important impact on our confidence in the estimate of effect and may change the estimate. **Low quality:** Further research is very likely to have an important impact on our confidence in the estimate of effect and is likely to change the estimate. **Very low quality:** We are very uncertain about the estimate. | | | | | |
| ^1^ without concealment ^2^ I²＞50% ^3^ RR>2 or RR<0.5 ^4^ total number events is less than 300 ^5^ RR>5 or RR<0.2 | | | | | |
